# Poor data stewardship will hinder global genetic diversity surveillance

**DOI:** 10.1073/pnas.2107934118

**Published:** 2021-08-17

**Authors:** Rachel H. Toczydlowski, Libby Liggins, Michelle R. Gaither, Tanner J. Anderson, Randi L. Barton, Justin T. Berg, Sofia G. Beskid, Beth Davis, Alonso Delgado, Emily Farrell, Maryam Ghoojaei, Nan Himmelsbach, Ann E. Holmes, Samantha R. Queeno, Thienthanh Trinh, Courtney A. Weyand, Gideon S. Bradburd, Cynthia Riginos, Robert J. Toonen, Eric D. Crandall

**Affiliations:** ^a^Department of Integrative Biology, Ecology, Evolution, and Behavior Program, Michigan State University, East Lansing, MI 48824;; ^b^School of Natural and Computational Sciences, Massey University, Auckland 0745, New Zealand;; ^c^Department of Biology, University of Central Florida, Orlando, FL 32816;; ^d^Department of Anthropology, University of Oregon, Eugene, OR 97403;; ^e^Moss Landing Marine Laboratories, California State University Monterey Bay, Moss Landing, CA 95039;; ^f^Marine Laboratory, University of Guam, Mangilao 96910, Guam;; ^g^Department of Integrative Biology, University of Texas at Austin, Austin, TX 78712;; ^h^Department of Evolution, Ecology, and Organismal Biology, The Ohio State University, Columbus, OH 43210;; ^i^Department of Natural Science, Hawaii Pacific University, Honolulu, HI 96813;; ^j^Department of Animal Science, University of California, Davis, CA 95616;; ^k^Department of Biological Sciences, Auburn University, Auburn, AL 36849;; ^l^School of Biological Sciences, The University of Queensland, Brisbane, QLD 4072, Australia;; ^m^Hawai‘i Institute of Marine Biology, University of Hawai‘i at Mānoa, Kāne‘ohe, HI 96744;; ^n^Department of Biology, Pennsylvania State University, University Park, Pennsylvania, PA 16802

**Keywords:** genomic, metadata, conservation, biodiversity, management

## Abstract

Genomic data are being produced and archived at a prodigious rate, and current studies could become historical baselines for future global genetic diversity analyses and monitoring programs. However, when we evaluated the potential utility of genomic data from wild and domesticated eukaryote species in the world’s largest genomic data repository, we found that most archived genomic datasets (86%) lacked the spatiotemporal metadata necessary for genetic biodiversity surveillance. Labor-intensive scouring of a subset of published papers yielded geospatial coordinates and collection years for only 33% (39% if place names were considered) of these genomic datasets. Streamlined data input processes, updated metadata deposition policies, and enhanced scientific community awareness are urgently needed to preserve these irreplaceable records of today’s genetic biodiversity and to plug the growing metadata gap.

Genomic data have never been more available. Researchers can now genotype thousands of loci or sequence whole genomes from virtually any species, and these data are deposited in open-access repositories. Although generated for diverse research purposes, much of these archived genomic data have immense reuse value for measuring genetic diversity—the raw material on which species’ health depends (1, 2). In principle, these data can provide time-stamped records for genetic diversity monitoring (3, 4) (supporting the goals of the United Nations Convention on Biological Diversity [CBD]) (5) and can be used to elucidate the evolutionary and ecological processes that shape biodiversity across the globe (6). Thus, raw genomic data in public repositories are inimitable historical resources—analogous to natural history museums—for the most fundamental level of biodiversity. However, reuse of genomic sequences also minimally requires information about the spatial and temporal context of the sampled organisms (7). Without appropriate archival practices that maintain links between genotypes, place, and time, these growing genomic resources will have limited real-world impact on genetic diversity surveillance.

To evaluate whether genomic data and spatiotemporal metadata are adequately archived, we conducted a structured search of publicly available data (*SI Appendix*, Appendix S1–S3 and *Supplementary Methods*) in the International Nucleotide Sequence Database Collaboration (INSDC) (8). Most scientific journals require authors to archive their genetic data in a permanent database, and the INSDC is the leading repository of raw genomic data. Data are submitted through one of three INSDC data centers—Japan’s DNA Data Bank of Japan, the European Molecular Biology Laboratory’s European Bioinformatics Institute, or the United States’ National Center for Biotechnology Information (NCBI) (which includes the original sequence repository GenBank)—and are propagated into the other two daily. We accessed the INSDC records through the NCBI portal. We focused on wild and domesticated species, because these are the most common targets for biodiversity studies. Whereas most studies describing spatial and temporal patterns in genetic diversity include wild populations (6, 9), the CBD prioritizes conserving domesticated species (and their wild relatives) and aspires to detect temporal trends in the genetic diversity of stocks and cultivars (5).

As of October 2020, the Sequence Read Archive (SRA) of the INSDC contained 600 terabytes (1.63 quadrillion base pairs) of genomic data representing over 16,700 unique wild and domesticated eukaryotic species and 327,577 individual organisms (BioSamples, Fig. 1) in 5,043 datasets (BioProjects). Alarmingly, we found that genomic records for only 14% of these individuals included the spatiotemporal metadata required for genetic diversity monitoring. After removing 562 domesticated species, we were left with 233,639 sequenced individuals from putatively wild populations in 3,903 datasets. Individuals in 17% of these datasets had geospatial coordinates, 41% had collection years, and only 14% had both. With manual effort, approximate geospatial context could be inferred for individuals in about half of these 3,093 datasets—51% had place names (e.g., Lake Mendota) and 66% had country names (Fig. 2*A* and *SI Appendix*, Appendix S3). Still only 38% had some location data and a collection year. Records from domesticated species had similar or more extreme levels of missing metadata compared to those from wild species (Fig. 2*A*).

**Fig. 1.**
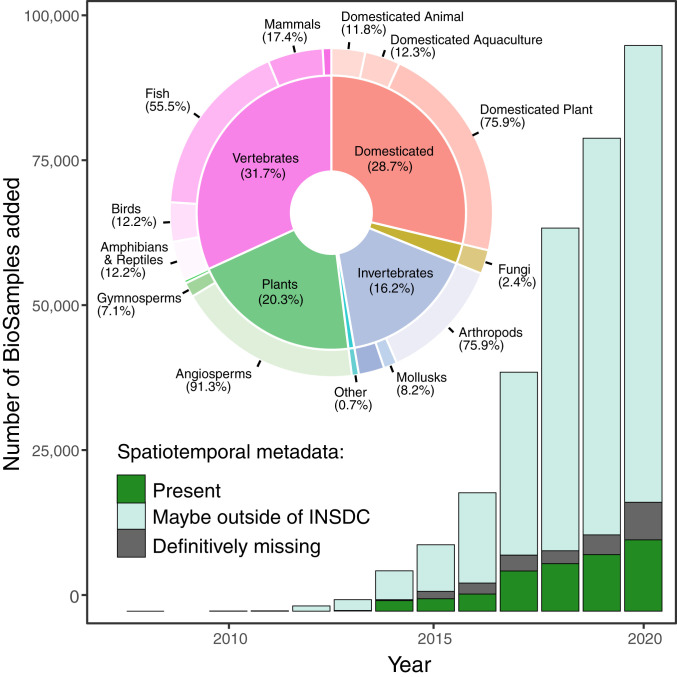
Genomic-level sequence data are being added to the INSDC at an exponential rate across eukaryotic taxa. Colors represent the status of spatiotemporal metadata (latitude/longitude and collection year) for each individual (BioSample, *n* = 327,577, see *SI Appendix*, Appendices S1–S3). (*Inset*) Taxonomic breakdown of BioSamples. Percentages in outer rings sum to corresponding inner-ring totals. Unlabeled inner-ring slices correspond to “other” for the outer-ring taxa.

**Fig. 2.**
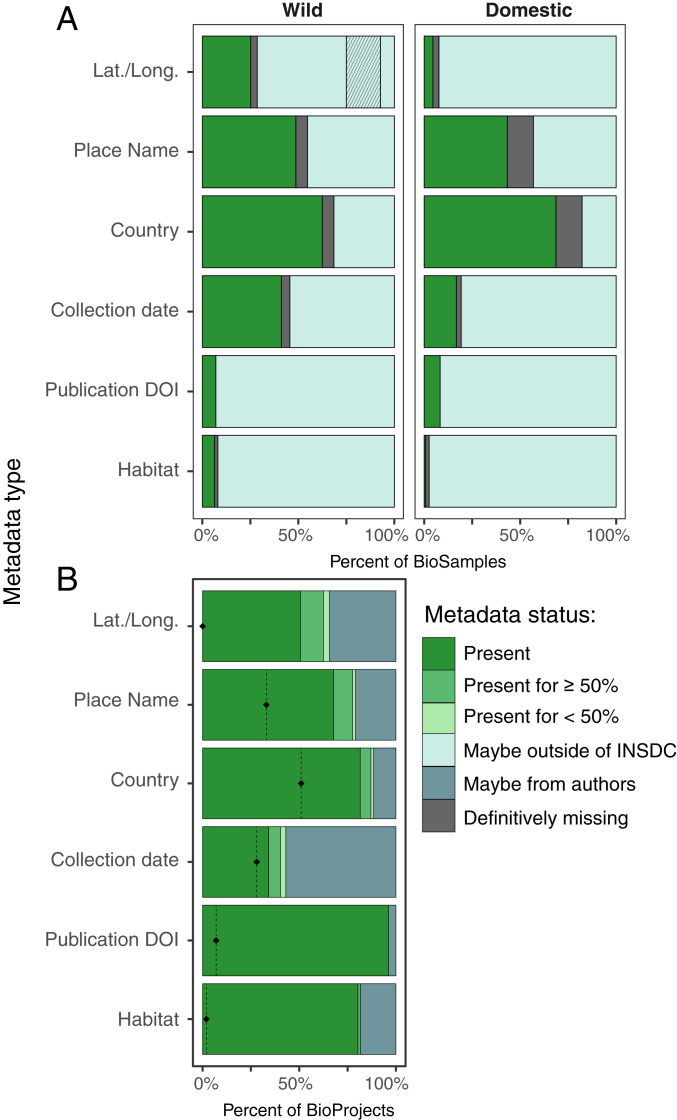
Most genomic-level sequence data in the INSDC lack critical metadata. (*A*) Status of metadata in the INSDC for wild and domesticated individuals (BioSamples, *n* = 327,577). Gray hashed box indicates datasets (BioProjects) with more than four wild individuals that lacked latitude/longitude and are addressed in *B* (*n* = 493). (*B*) Status of metadata for records inside hashed box in *A* after augmenting with metadata from associated publications. *Left* of black diamonds = present in INSDC.

To explore whether the levels of missing metadata that we report for putatively wild populations were inflated by including nonwild individuals, we tested how accurately our filters identified wild individuals. We randomly subsampled 200 datasets from the 3,093 datasets programmatically identified as “wild” and read their associated scientific publications. Based on this subsample, 70% of the datasets identified as wild by our filters were in fact from wild populations. Spatiotemporal metadata were present for only 13% (bootstrapped 95% CI: 6 to 20%) of these datasets, suggesting the 14% we report for the 3,093 putatively wild datasets is representative. Adding a searchable INSDC field that identifies wild-collected individuals would greatly benefit future genetic diversity syntheses and monitoring efforts.

We further investigated whether missing spatiotemporal metadata could be manually recovered from sources external to the INSDC. We prioritized 848 genomic datasets (representing 94,416 individuals) deemed relevant for conservation monitoring, because they each described more than four putatively wild individuals. We located published scientific papers describing 739 (of 852) datasets. By reading these papers, we determined that 493 datasets (representing 57,396 individuals) reported genetic diversity for wild populations, and we increased metadata coverage for each category (Fig. 2*B*). After these manual efforts, individuals in 63% of these datasets had geospatial coordinates, 40% had collection years, and 33% had both (39% if any type of location data were considered).

In summary, most depositions in the SRA lack sufficient spatiotemporal metadata to enable future reuse and genetic diversity monitoring. Even time-consuming manual efforts to recover these data (∼2,000 human hours here) are only partially successful. Working directly with individual authors is the only remaining strategy to potentially recover these missing metadata (e.g., from personal files or memory) and these missing metadata become increasingly difficult to recover with time since deposition (10). In cases where metadata were never collected or lost, the genomic data may simply be unusable for future analyses. Assuming a sequencing cost of $50/individual, the lost investment from missing spatiotemporal metadata identified in this effort totals tens of millions of US dollars, and this amount will likely grow exponentially each year (Fig. 1). Moreover, this estimate ignores the cost of fieldwork and sampling and the fact that most past timepoints cannot be resampled.

The genetics community has long championed open-data publication. The INSDC databases originated in the early 1980s (8), and a combination of top-down mandates and recognition of open-data benefits helped ingrain open-data values in the research community. Only since 2008, however, were the Minimum Information about any Sequence (MIxS) metadata standards formulated (11), which encouraged the community to provide metadata about what (taxonomy), where (georeferences and habitat type), when (collection date), how (sampling and sequencing protocols), and by whom a sample was collected. Initiatives from journals and funders such as the Joint Data Archival Policy have improved genetic metadata quality (7). But, our assessment of the INSDC highlights a gap between which metadata should be collected and archived and which metadata are collected and archived.

Solutions to the metadata gap require understanding of why metadata are missing. In some cases metadata are not collected, as this contextual information is nonessential for the original study. In most cases, however, the intent of the original study suggests that metadata should exist, but researchers depositing the genomic data have either not followed the FAIR Guiding Principles for data stewardship (data should be findable, accessible, interoperable, and reusable; ref. 12) or have misfiled their metadata within the INSDC fields (Fig. 2*B*). Although the INSDC was not designed to be a metadatabase for genetic diversity studies, and issues of data integrity will always persist in data repositories of this size (13), repositories have a responsibility to help researchers be compliant with community standards (sensu ref. 14). Simpler deposition protocols would encourage researchers to link spatiotemporal metadata with sequence data of individuals. The metadata that we recovered, for example, will be accessioned to the Genomics Observatories Metadatabase (GEOME; ref. 15), which provides a user-friendly portal for researchers to upload MIxS-compliant, FAIR metadata (to GEOME), and genomic data (to the INSDC SRA). From GEOME, these metadata can easily be cross-walked into INSDC. Incentivizing changes in researcher behavior may additionally require journals and funders to mandate the deposition of spatiotemporal metadata when it is relevant to reuse the genomic data, and for data publications to be rewarded appropriately in hiring, promotion, and tenure decisions. We urge journals to join *Molecular Ecology* in encouraging authors to link spatiotemporal metadata to genetic sequence data generated for wild species and domesticated species where available (16). While the initial success of GenBank relied on maturing community consensus around the value of open data, today’s increasing rate of biodiversity loss (9) makes ongoing spatiotemporal metadata loss an urgent community issue.

We join others in calling for ambitious goals to safeguard genetic diversity (3, 7, 17) and the knowledge structures that will support this goal. Common to proposed genetic diversity monitoring agendas is a shared vision whereby agile pipelines would intake raw genomic data and produce outputs that directly inform conservation policies and decisions. Yet, without appropriate archival genomic data that include the spatiotemporal metadata, crucial information will be unavailable to such pipelines, and researchers will be unable to monitor genetic biodiversity or to reconstruct past baselines.

Our critical evaluation of whether publicly available genomic data could be used for meaningful biodiversity analyses and assessments shows that most records fall short. The identified metadata gap represents an irreplaceable loss of historical details. In 2019 alone, the SRA grew by 50%, with the addition of trillions of base pairs of DNA sequence added per day. Meanwhile the world’s sixth mass extinction event is underway with 35,000 species now listed as endangered (i.e., The International Union for Conservation of Nature’s Red List of Threatened Species, https://www.iucnredlist.org/en). Now is the time to plug this metadata gap for the most foundational layer of biodiversity. Our future ability to study, monitor, and conserve all levels of biodiversity depends on it.

## Supplementary Material

Supplementary File

## Data Availability

All study data are included in the article and/or supporting information. Previously published data were used for this work (thousands of INSDC SRA records, ref. 8). A list of the INSDC records and associated code are stored on BitBucket at https://bitbucket.org/toczydlowski/status_of_insdc_genomic_metadata/src/master/.
