# Supplementary material for: Poor data stewardship will hinder global genetic diversity surveillance
Source: Proc Natl Acad Sci U S A. 2021 Aug 17;118(34):e2107934118. doi: 10.1073/pnas.2107934118 (PMC8403888; doi:10.1073/pnas.2107934118)
Supplement: Supplementary File [file pnas.2107934118.sapp.pdf]

PNAS
www.pnas.org

Supplementary Information for

Poor data stewardship will hinder global genetic diversity surveillance

Rachel H. Toczydlowski[1*], Libby Liggins[2], Michelle R. Gaither[3], Tanner J. Anderson[4], Randi L. Barton[5], Justin T. Berg[6], Sofia G. Beskid[7], Beth Davis[5] , Alonso Delgado[8], Emily Farrell[3], Maryam Ghoojaei[3], Nan Himmelsbach[9], Ann E. Holmes[10], Samantha R. Queeno[4], Thienthanh Trinh[3], Courtney A. Weyand[11], Gideon S. Bradburd[1], Cynthia Riginos[12], Robert J. Toonen[13], Eric D. Crandall[14†]

[1]Department of Integrative Biology - Ecology, Evolution, and Behavior Program, Michigan State University, East Lansing, MI 48824.
[2]Massey University, Auckland, Aotearoa.
[3]Department of Biology, University of Central Florida, Orlando, FL 32816.
[4]Department of Anthropology, University of Oregon, Eugene, OR 97403.
[5]Moss Landing Marine Laboratories, California State University Monterey Bay, Moss Landing, CA 95039.
[6]Marine Laboratory, University of Guam, Mangilao, Guam 96910.
[7]Department of Integrative Biology, University of Texas at Austin, Austin, TX 78712.
[8]Department of Evolution, Ecology, and Organismal Biology, Ohio State University; Columbus, OH 43210.
[9]Department of Natural Science, Hawaii Pacific University, Honolulu, HI 96813.
[10]Department of Animal Science, University of California-Davis, Davis, CA 95616.
[11]Department of Biological Sciences, Auburn University, Auburn, AL 36849.
[12]School of Biological Sciences, The University of Queensland, Brisbane.
[13]HIMB, University of Hawai‘i at Mānoa, Kāne‘ohe, HI 96744.
[14]Department of Biology, Pennsylvania State University, University Park, Pennsylvania, PA 16801.

[*]Rachel H. Toczydlowski
Email: rhtoczyd@msu.edu
[†]Eric D. Crandall
Email: ecrandall@psu.edu

This PDF file includes:

Supplementary text
Appendix S1: Overview of genetic sample search and filtering procedure
Appendix S2: Exact terms used to locate and filter INSDC datasets
Appendix S3: Description of the INSDC attributes that form the basis of reported metadata
SI References

Other supplementary materials for this manuscript include the following:

Dataset S1

Supplementary Information Text

Supplementary Methods

Locating and filtering genomic datasets and associated metadata in the INSDC —
We used the following search strategy to locate and download metadata associated with genetic records in the International Nucleotide Sequence Database Consortium (INSDC). Our goal was to identify genomic-level DNA sequence data (i.e. RADseq type data and whole genome data) for non-human, non-viral, non-metagenomic, non-bacterial, and non-model organism individuals. We searched the BioProject database of the INSDC on October 19, 2020 using the below query (see Appendix S1). We then located and downloaded all metadata associated with these BioProjects in the PubMed, Taxonomy, BioSample, and Sequence Read Archive (SRA) INSDC databases. BioProjects are the highest level of project organization in INSDC and commonly correspond to a published study. The PubMed database contains published papers. Taxonomy contains taxonomic information for the individual samples. BioSamples generally correspond to the individual plant, animal, etc. that the tissue came from to generate (genetic sequence) data. The SRA is the finest level of organization and contains metadata associated with individual FASTQ/A genetic sequence files. We used the entrez_search() function to locate entries in INSDC and entrez_summary() and/or entrez_fetch() to download the associated metadata from the rentrez R package (V1.2.3, (1)). We used relevant INSDC metadata to remove as many human, viral, bacterial, metagenomic, and environmental DNA samples as possible (see Appendix S2 for exact filters). We retained samples that were RAD-seq type sequencing or full genome of nuclear DNA as these methods are commonly used to derive population genomic estimates of genetic diversity.

After applying as many filters as were relevant within the INSDC metadata, we developed a list of taxonomic names for taxa that are likely not from natural, wild populations (Data S1). We targeted three main categories of species (or genera): 1) human pathogens and their vectors, 2) model organisms, and 3) domesticated species. We used this list as a final filtering step to retain species most likely to be relevant for genetic diversity monitoring of wild populations. We recognize that this list cannot be exhaustive and that a significant grey area exists between domesticated and wild populations. We generated the lists of human pathogens, model organisms, and domestic species using the following sources (Data S1). For human pathogens, we used the organism table from VEuPathDB (2), a consortium database for human eukaryotic pathogens and vectors (accessed Jan 25, 2021), and augmented this with a Wikipedia list of infectious diseases (3). We generated the list of model organisms using Wikipedia lists of model organisms (4). We built sub-lists for domesticated animals, plants, and aquaculture. For domesticated plants, we started from supplemental table S1 of Khoury et al. (5), which in turn was compiled from a standard list of crops reported in the FAOSTAT database (6). We augmented this list from the United States Agricultural Research Service Germplasm Resources Information Network's (7) listing of World Economic Plants (8) for Human Food and Lawn and Turf categories, and the Wikipedia list of domesticated plants (9). For domesticated animals, we used Table 1A1 from the State of Livestock Diversity Annex to the FAO's Second Report on the State of the World's Animal Genetic Resources (10), and augmented this list from Wikipedia's list of domesticated animals (11). For domesticated aquaculture species, we used FAO's, the state of the world's fisheries and aquaculture (12). We created one master list (Data S1) using the R package *dplyr* (13) and removed duplicated binomials within categories (citing each to the most authoritative source). We hand-edited this list to remove species that we judged to have significant natural populations (e.g. we removed Three-spined stickleback, *Gasterosteus aculeatus*, from the list of model organisms), in keeping with our desire to comment on metadata from potentially natural populations. In cases where we judged an entire genus to fall into one of the three categories, we retained only the genus name (e.g. *Citrus*).

We removed BioSamples with taxonomic names that matched those in the model organism and human pathogen categories (the taxonomic names of BioSamples are the most heavily curated by at least NCBI staff) using a grepl() search command. We retained but flagged BioSamples that

matched our domesticated species list so we could explore the metadata status for putatively wild individuals from natural populations and domesticated species separately. We defined putatively wild individuals as BioSamples that remained after applying all filters in Appendix S1 (including "not domesticated"; *N* = 233,644 BioSamples).

We extracted specific metadata categories of interest from the INSDC records using R (see Appendix 3 for exact pieces of metadata extracted and reported on). We note that the same type of metadata can be stored under multiple field names, or under unintuitive field names, in INSDC. We searched through all possible metadata returned by our rentrez searches and online in the INSDC interface for a small subset of datasets to identify columns containing relevant metadata. Even so, there are likely still instances where metadata did exist for a sample but was coded as missing in this survey because it occurred in a different field. For example, BioProject PRJNA214891 did not contain a field for collection date (and was thus scored as missing this information) but did contain a field called death_date, which is presumably the same or similar to the collection date. We classified metadata as "definitively missing" (Fig. 1,2) if the value for in the metadata field was any non-case sensitive form of: missing, not collected, not available, not applicable, unknown, unspecified, ?, -, or null. If the metadata field was blank, we coded the metadata as "maybe outside of INSDC". We coded fields with values as "present". We use "spatiotemporal" throughout the paper to mean geospatial coordinates and collection year.

Calculating levels of present/absent metadata —
We first assessed whether it was more appropriate to calculate levels of present/absent metadata using BioSamples or BioProjects as the unit of analyses. The types of metadata we report on (e.g. geospatial coordinates and collection year) correspond to the level of the individual organism sampled. However, calculating the percentages of missing metadata using individuals as the unit of analysis could lead to pseudoreplication if researchers commonly collect and report metadata for all or none of the samples in a dataset. We confirmed this to be the case for the 5,043 BioProjects that we report on (domesticated and putatively wild individuals). Only 13% of these BioProjects had mixed present/absent metadata among their BioSamples in at least one of the metadata categories we highlight: geospatial coordinates, collection year, place name, and country. This percentage was 14% for the subset of BioProjects from putatively wild individuals and 9% for the subset of BioProjects from species in our domesticated list. Given metadata were absent or present for all BioSamples in the majority of BioProjects, we present percentages of present/absent metadata calculated using BioProject as the unit of analysis. We counted "mixed" BioProject as having metadata present if at least 50% of its BioSamples had that type of metadata present and absent if fewer than 50% of its BioSamples had metadata. Levels of present metadata calculated at the level of BioSamples were at most 8% higher (geospatial coordinates for putatively wild individuals) and at least 4% lower (country names for putatively wild individuals) than the values we report at the BioProject level. BioProjects with spatiotemporal metadata present had on average 80 ± 8 (*se*) BioSamples as compared to 57 ± 3 (*se*) BioSamples in BioProjects that lacked spatiotemporal metadata.

Evaluating ability of filters to identify wild individuals —
To evaluate how well the above filters identify individuals from wild populations, we took a random sample of 200 BioProjects (with no constraints on the number of BioSamples per BioProject) from the 3,903 BioProjects identified as putatively from wild populations. We then searched scholarly databases for associated scientific publications for these 200 BioProjects and read through the methods to determine whether at least 50% of sequenced samples came from wild populations. From this survey, we determined that 139 of the 200 pre-filtered BioProjects had over 50% BioSamples from wild populations; 14 BioProjects described domesticated species not in our list, 40 were from laboratory or brood stocks, 2 were eukaryotic human pathogens not in our list, 3 were not relevant to biodiversity for other reasons, and 2 BioProjects each contained a single BioSample whose provenance could not be determined. We then calculated the level of spatiotemporal metadata present for the subset of 139 wild datasets and compared it to the level for the 61 datasets mis-identified by our filters as wild, and the level we calculated using all 3,903 putatively wild datasets. We calculated a bootstrapped confidence interval for the levels of

present spatiotemporal metadata in the 139 wild and 61 non-wild datasets by taking 100 random draws of a sample size 50% of the total pool of each type of dataset. Importantly, these levels of present spatiotemporal data were all highly similar - 14% for the 3,903 total putatively wild datasets, 13% (95% CI: 6-20%) for the 139 wild datasets in the random subset, and 14% (95% CI: 3-23%) for the 61 datasets mis-identified as wild in the random subset. Although our filters did not perfectly identify only studies from wild populations that are relevant to genetic diversity monitoring, the inclusion of non-relevant studies does not appear to be strongly affecting the levels of missing spatiotemporal metadata that report.

Searching for associated metadata outside of INSDC —
We used the following protocol to search for and retrieve metadata from associated published papers and other online sources for relevant BioProjects that did not contain spatial coordinates for genetic samples. We note that we only searched for metadata outside of INSDC for relevant samples that were published before October 28, 2019, the date that we first searched INSDC. We first filtered the BioProjects containing samples from presumably wild populations (*N* = 3,903) to those that contained at least 5 unique BioSample IDs. Datasets with fewer than 5 individuals are likely to be of less use in monitoring population- and species-level genetic diversity. In addition to having small sample sizes, datasets with fewer than 5 individuals are also more likely to represent datasets initially collected for purposes other than assessing genetic diversity of wild populations - for example, building genetic maps, QTL-mapping, genome assembly, and GWAS. However, levels of missing metadata calculated for all putatively wild BioProjects, only those with fewer than 5 BioSamples per BioProject, and only those with 5 or more BioSamples were all within 5% of each other for geospatial coordinates, collection year, place name, and country name. Regardless of the general relevance of BioProjects with few BioSamples to genetic diversity monitoring, they have similar levels of metadata reported in the INSDC as those with more BioSamples.

When no published paper was explicitly linked to a BioProject in INSDC, (the vast majority of cases, see Figure 2A), we performed keyword searches in online search engines (e.g. Google Scholar) to try to locate associated published papers. We searched for various combinations of taxon names, keywords from the BioProject description and title, submitter names and institutions, INSDC project identifiers (e.g. the PRJ BioProject ID, which is often published in the Data Availability statements of associated papers), and funding information that were associated with each BioProject in INSDC. We determined a BioProject to be relevant to our efforts if the sequenced samples did not come from human pathogens, model organisms, lab or brood stocks, or domesticated species (see above). When BioProjects included a mix of samples from wild and non-wild categories, we retained the BioProject if at least 50% of the samples and/or sampled sites came from wild populations. We made these decisions by reading the published scientific paper/s associated with the BioProject. When we could not confidently match the material sample identifiers in the SRA to relevant metadata in a publication, we coded materialSampleID = FALSE (but other provided categories as TRUE). When MIxS terms env_broad_scale (Habitat), env_local_scale (microhabitat) and env_medium (environmental medium) were not explicitly stated in the paper or online resource but could confidently be assigned by the metadata curator based on common knowledge (e.g. tuna live in the oceanic (env_broad_scale) pelagic zone (env_local_scale), displacing sea water (env_medium), these categories were coded as TRUE. We note that the work to retrieve metadata external to INSDC was conducted by several graduate students (authors on this paper) via a virtual datathon while their own research was upended by the COVID-19 pandemic. This protocol and the virtual datathon will be further elaborated in an upcoming manuscript.

Appendix S1
Overview of genetic sample search and filtering procedure

184,155 total BioProjects in INSDC on 10/19/2020

Search BioProject database for all BioProjects that are:
Non-human, non-viral, non-metagenomic, and non-bacterial
32,162 BioProjects

Remove BioProjects related to:
Gene expression, RefSeq, targeted loci, metagenomic, metabarcoding, exome, microbial
19,495 BioProjects retained

Download metadata from the Sequence Read Archive (SRA) for all sequence records in each of the above BioProjects.
948,446 SRA sequence records

Retain SRA records with library strategies:
WGS, WCS, WGA, RAD-Seq, OTHER
(removing: AMPLICON, ATAC-seq, Bisulfite-Seq, ChIA-PET, ChIP-Seq, CLONE, CLONEEND, CTS, DNase-Hypersensitivity, EST, FAIRE-seq, Hi-C, FINISHING, FL-cDNA, MBD, MeDIP, miRNA-Seq, MNase-Seq, MRE-Seq, ncRNA-Seq, POOLCLONE, RIP-Seq, RNA-Seq, SELEX, Synthetic-Long-Read, Targeted-Capture, TetheredChromatinConformationCapture, Tn-Seq, WXS)
523,537 sequence records retained

Merge these SRA records to the BioProject records using PRJ BioProject accession number.

Remove BioProjects that didn't return any hits to the SRA database.
7,314 BioProjects retained

View all taxonomic IDs assigned to current list of sequence records in NCBI common tree online tool.
Remove sequences with a species identified as:
Synthetic, metagenome, unidentified, bacteria, environmental, viral
505,609 sequences from 432,079 BioSamples from 6,741 BioProjects retained

Download metadata from the BioSample database associated with each of the above sequence records.

Download metadata from the PubMed database associated with each BioProject publication ID.

Remove BioSamples from species that are (common) model organism, human pathogen:
(using list: nonWildSpecies_final_sources.tsv;
see Supplementary Methods for details)
380,416 sequences from 327,582 BioSamples from 5,043 BioProjects retained

Denote BioSamples from domesticated species:
(using list: nonWildSpecies_final_sources.tsv;
see Supplementary Methods for details)
presumably wild: 268,384 sequences from 233,644 BioSamples, from 3,903 BioProjects

Appendix S2
Exact terms used to locate and filter INSDC datasets

The exact search terms and filters that we used to identify the INSDC datasets analyzed in this publication follow. Note that we use "!=" below to denote "does not equal".

Initial BioProject search:
```
"scope multiisolate"[Filter] OR "scope multispecies"[Filter] OR "scope
other"[Filter] NOT "org human"[Filter] NOT "org archaea"[Filter] NOT
"org viruses"[Filter] NOT "org bacteria"[Filter] NOT
"metagenome"[Filter] NOT "targeted locus loci"[Filter] NOT "clone
ends"[Filter] NOT "metagenomic assembly"[Filter] NOT "transcriptome
gene expression"[Filter] NOT "proteome"[Filter] NOT
"epigenomics"[Filter] NOT "exome"[Filter] NOT "bioproject
protein"[Filter] NOT "bioproject gds"[Filter] NOT "phenotype
genotype"[Filter] NOT "metagenome"[All Fields] NOT "map"[Filter]
```

BioProject filters:
project_data_type !=
```
RefSeq Transcriptome or Gene expression
Targeted loci cultured
RefSeq Genome
RefSeq Genome sequencing and assembly
RefSeq Other
RefSeq Targeted Locus (Loci)
```

project_target_material !=
```
Phenotype
Proteome
Purified Chromosome
Reagent
Transcriptome
Targeted loci environmental
```

project_target_capture !=
```
Clone Ends
Exome
```

organism_name does not contain:
```
metagenome
```

project_title does not contain:
```
metagenome, Metagenome, Metagenomic, metagenomic, metabarcoding
transcriptome, Transcriptome, Transcriptomic
RNA
16S, 16s, 18S, 18s
CRISPR
gene expression, Gene Expression, Gene expression"
viral
microbiome, Microbiome, microbial, microbiota
bacteria, Bacteria
methylation
Plasmodium falciparum
```

SRA filters:
library_strat =

```
RAD-Seq
WCS
WGA
WGS
OTHER
```

<u>scientific_name !=</u>

```
metagenome, Metagenome
```

<u>taxid !=</u>

```
2293429
1440148
32630
1427524
81077
32644
496923
77133
669196
2282120
668369
727
2590021
28448
272943
2021969
527802
477819
562
227
83333
316407
703612
224308
1229511
1126252
1126251
```

Note that the above taxonomic IDs that we filtered out represent synthetic DNA, environmental DNA, metagenomic DNA, and bacterial DNA. We identified these "organisms" by viewing the taxonomic IDs associated with the sequence data in our data frame in the INSDC phylogenetic common tree online tool.

Taxonomy filters:
<u>division !=</u>

```
Environmental samples
Bacteria
Viruses
```

BioSample filters:
<u>organism_name !=</u>
human pathogens and model organisms listed in: nonWildSpecies_final_sources.tsv
(see Supplementary Methods)

Appendix S3
Description of the INSDC attributes that form the basis of reported metadata

Example interpretation for first entry below:
The piece of metadata that we defined as latitude and longitude in this paper came from using the entrez_summary() function of the rentrez R package to search the INSDC database BioSample, which returned an attribute called sampledata, which contained longitude and latitude data stored under an attribute with the display name “latitude and longitude”.

Latitude and longitude (Lat. + long.)
`entrez_summary(db = "biosample") → sampledata → display_name= "latitude and longitude"`

Publication DOI
Deemed present if there was a value in at least one of the following attributes:
`entrez_fetch(db = "bioproject", rettype = "xml") → Publication\ id`
AND/OR
`entrez_summary(db = "pubmed") → articleids → doi`

Place name
`entrez_summary(db = "biosample") → sampledata → display_name="geographic location" → value present after ":"`

Country
`entrez_summary(db = "biosample") → sampledata → display_name="geographic location" → value present before ":"`

Collection date
`entrez_summary(db = "biosample") → sampledata → display_name="collection date"`

Material sample ID
Deemed present if there was a value in at least one of the following attributes:
`entrez_summary(db = "sra") → expxml → LIBRARY_NAME`
AND/OR
`entrez_summary(db = "biosample") → sampledata → display_name="sample name"`
AND/OR
`entrez_summary(db = "biosample") → identifiers → display_name=Sample name`

Permit ID
Could not locate this attribute in INSDC

Habitat
`entrez_summary(db = "biosample") → sampledata → display_name="broad-scale environmental context"`

Spatiotemporal
Deemed present if there was a value present for both **latitude and longitude** AND **collection date**

Dataset S1 (separate file).

nonWildSpecies_final_sources.tsv

List of human pathogens, model organisms, and domesticated species that we assembled and used to identify BioSamples from presumably wild populations.

SI References

1. D. J. Winter. rentrez: an R package for the NCBI eUtils API. *The R Journal* 9, 520-526 (2017).
2. C. Aurrecoechea, A. Barreto, E. Y. Basenko, J. Brestelli, B. P. Brunk, S. Cade, K. Crouch, R. Doherty, D. Falke, S. Fischer, B. Gajria, O. S. Harb, M. Heiges, C. Hertz-Fowler, S. Hu, J. Iodice, J. C. Kissinger, C. Lawrence, W. Li, D. F. Pinney, J. A. Pulman, D. S. Roos, A. Shanmugasundram, F. Silva-Franco, S. Steinbiss, C. J. Stoeckert, D. Spruill, H. Wang, S. Warrenfeltz, J. Zheng, EuPathDB: the eukaryotic pathogen genomics database resource. *Nucleic Acids Res*. 45, D581–D591 (2017).
3. List of Infectious Diseases. (n.d.). In Wikipedia. Retrieved January 25, 2021, from https://en.wikipedia.org/wiki/List_of_infectious_diseases.
4. Model Organisms. (n.d.). In Wikipedia. Retrieved January 25, 2021, from https://en.wikipedia.org/wiki/Model_organism and https://en.wikipedia.org/wiki/List_of_model_organisms.
5. C. K. Khoury, H. A. Achicanoy, A. D. Bjorkman, C. Navarro-Racines, L. Guarino, X. Flores-Palacios, J. M. M. Engels, J. H. Wiersema, H. Dempewolf, S. Sotelo, J. Ramírez-Villegas, N. P. Castañeda-Álvarez, C. Fowler, A. Jarvis, L. H. Rieseberg, P. C. Struik, Origins of food crops connect countries worldwide. *Proc. R. Soc. B*. 283, 20160792 (2016).
6. FAO. 2015 FAOSTAT. Rome, Italy: Food and Agriculture Organization of the United Nations. See http://faostat3.fao.org/.
7. Germplasm Resources Information Network [Internet]. Beltsville (MD): United States Department of Agriculture, Agricultural Research Service. Available from: http://www.ars-grin.gov/.
8. J. H. Wiersema, B. Léon, World Economic Plants (Taylor & Francis, Boca Raton, FL, 2013).
9. List of Domesticated Plants. (n.d.). In Wikipedia. Retrieved January 25, 2021, from https://en.wikipedia.org/wiki/List_of_domesticated_plants.
10. B. D. Scherf, D. Pilling, Commission on Genetic Resources for Food and Agriculture, The second report on the state of the world's animal genetic resources for food and agriculture (2015; http://www.fao.org/3/a-i4787e.pdf).
11. List of Domesticated Animals. (n.d.). In Wikipedia. Retrieved January 25, 2021, from https://en.wikipedia.org/wiki/List_of_domesticated_animals.
12. FAO, Ed., The state of the world's fisheries and aquaculture (Rome, 2018), The state of world fisheries and aquaculture.
13. H. Wickham, R. François, L. Henry, K. E. Müller, dplyr: A Grammar of Data Manipulation. R package version 1.0.2. (2020; https://CRAN.R-project.org/package=dplyr).
